# Quiescence-inducing neurons-induced hypometabolism ameliorates acute kidney injury in a mouse model mimicking cardiovascular surgery requiring circulatory arrest

**DOI:** 10.1016/j.xjon.2022.11.001

**Published:** 2022-11-08

**Authors:** Shoichi Kyo, Kozue Murata, Masahide Kawatou, Kenji Minatoya, Genshiro A. Sunagawa, Hidetoshi Masumoto

**Affiliations:** a Department of Cardiovascular Surgery, Graduate School of Medicine, Kyoto University, Kyoto, Japan; b Clinical Translational Research Program, RIKEN Center for Biosystems Dynamics Research, Kobe, Japan; c Institute for Advancement of Clinical and Translational Science, Kyoto University Hospital, Kyoto, Japan; d Laboratory for Molecular Biology of Aging, RIKEN Center for Biosystems Dynamics Research, Kobe, Japan; e Laboratory for Hibernation Biology, RIKEN Center for Biosystems Dynamics Research, Kobe, Japan

**Keywords:** circulatory arrest, acute kidney injury, hibernation, Q neurons-induced hypometabolism, AAV8, adeno-associated virus 8, AKI, acute kidney injury, CH, control hypothermia, CN, control normothermia, CNO, clozapine-N-oxide, DHCA, deep hypothermic circulatory arrest, iCre, codon-improved Cre recombinase, NGAL, neutrophil gelatinase-associated lipocalin, Q neurons, quiescence-inducing neurons, QIH, quiescence-inducing neurons-induced hypometabolism, QH, quiescence-inducing neurons-induced hypometabolism-ready hypothermia, QN, quiescence-inducing neurons-induced hypometabolism-ready normothermia, QRFP, pyroglutamylated RFamide peptide, T_A_, ambient temperature, T_B_, body temperature, Vo_2_, the rate of oxygen consumption

## Abstract

**Objectives:**

Acute kidney injury is a serious complication after cardiovascular surgery requiring circulatory arrest. It is reported that mice can be induced into a hibernation-like hypometabolic state by stimulating a specific neuron located at the hypothalamus (quiescence-inducing neurons-induced hypometabolism [QIH]). Here, we investigated the efficacy of QIH for the amelioration of acute kidney injury in an experimental circulatory arrest using a transgenic mouse model.

**Methods:**

We genetically prepared mice in which QIH can be conditionally induced (QIH-ready mice). Mice were divided into 4 groups (n = 6 for each): QIH-ready normothermia (QN), QIH-ready hypothermia (QH), control normothermia (CN), and control hypothermia (CH). After induction of QIH, left thoracotomy and descending aorta crossclamping were conducted. After reperfusion, we collected kidneys and evaluated histologic changes and serum biochemical markers, specifically neutrophil gelatinase-associated lipocalin and cystatin C, indicating early kidney injury.

**Results:**

Normothermia showed higher tubular injury scores than those in hypothermia (QN vs QH [*P* = .0021] and CN vs CH [*P* < .001]). QN exhibited lower neutrophil gelatinase-associated lipocalin and cystatin C levels than those in CN (neutrophil gelatinase-associated lipocalin: CN vs QN: 1.51 ± 0.71 vs 0.82 ± 0.32; *P* = .0414 and cystatin C: 1.48 ± 0.39 vs 0.71 ± 0.26; *P* = .0015). There was no significant difference between QN and QH.

**Conclusions:**

QIH partly ameliorated acute kidney injury in a mouse ischemia model even in normothermia. QIH might be a promising approach to achieving sufficient kidney protection without hypothermic circulatory arrest in the future.

## Graphical abstract

**Figure:**
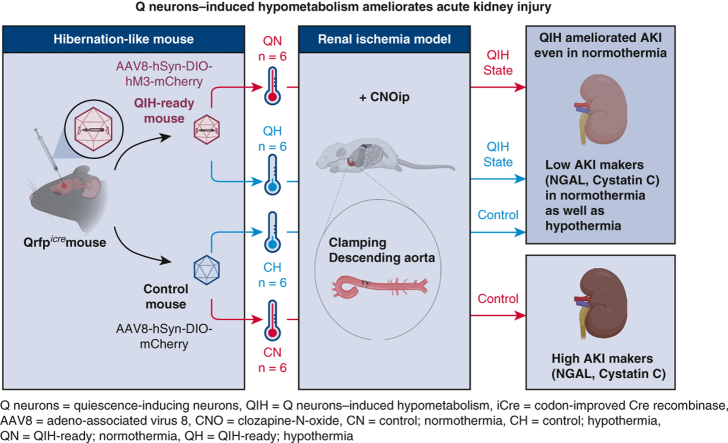

**Figure:**
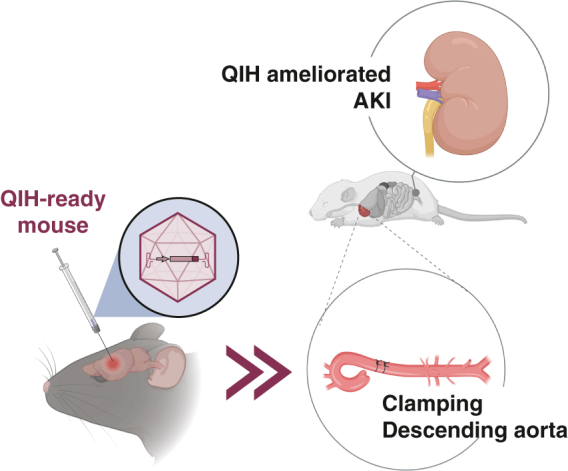
Q neurons-induced hypometabolism can ameliorate AKI in a renal ischemia model.

**Central Message**

Q neurons-induced hypometabolism ameliorates acute kidney injury mediated by ischemia even in normothermia in a mice model mimicking cardiovascular surgery requiring circulatory arrest.

**Perspective**

We have experimentally validated that Q neurons-induced hypometabolism (QIH) can protect the kidneys from acute kidney injury (AKI) mediated by ischemia in cardiovascular surgeries requiring circulatory arrest even in normothermia. QIH may contribute to establishing a new strategy to prevent AKI in cardiovascular surgeries requiring circulatory arrest without deep hypothermia in the future.

Acute kidney injury (AKI) is a major complication after cardiovascular surgery requiring circulatory arrest and is associated with increased short-term and long-term mortality. The incidence of AKI after aortic surgery requiring circulatory arrest is reported to be approximately 5% to 50%, which is higher than those of coronary artery bypass surgeries or valve surgeries^1^, ^2^, ^3^; indicating that circulatory arrest and associated kidney ischemia is a cause of the AKI.

It is well recognized that tissue oxygen consumption decreases in accordance with the reduction of body temperature, and the advantages of the reduction of body temperature in resuscitative medicine in cases of cardiopulmonary arrest or brain death are numerously reported since the 1950s.^4^, ^5^, ^6^ In the field of cardiovascular surgery requiring circulatory arrest, induction of hypometabolism by deep hypothermia below 20 °C is broadly applied since the 1970s for organ protection during the circulatory arrest.^7^ However, due to the tendency of postoperative coagulopathy and platelet dysfunction mediated by hypothermia,^8^ deep hypothermic circulatory arrest (DHCA) is associated with increased postoperative bleeding and blood transfusion requirement.^9^ Furthermore, DHCA is a cause of prolonged surgical duration, which may increase the risk of postoperative infectious complications,^10^^,^^11^ which encouraged surgeons and researchers to find an alternative to achieve sufficient organ protection during cardiovascular surgeries requiring circulatory arrest.

It is well known that some mammals, such as squirrels, can undergo a state of decreased physiological activity associated with reduced body temperature and metabolic activities called hibernation or torpor.^12^^,^^13^ The hibernators are capable of living in low body temperature and metabolic state under extremely reduced food intake and energy production.^12^^,^^14^ The fundamental of hibernation is the resistance to the hypometabolic state, or the mechanism allowing the animals to stay healthy even with a lower oxygen supply. Mice are recognized as a nonhibernator; however, our recent research revealed that mice can enter a hibernation-like state by stimulating pyroglutamylated RFamide peptide (QRFP)-containing neurons located at the hypothalamus (quiescence-inducing neurons [Q neurons]).^15^ The physiological function of Q neurons is not fully documented, yet, we found that excitation of Q neurons induces Q neurons–induced hypometabolism (QIH), a multiday hypometabolic state resembling hibernation. Moreover, we found that QIH mice exhibit decreased heart and respiratory rates associated with an extremely reduced rate of oxygen consumption (Vo_2_).^15^ This discovery indicates that nonhibernators, including humans, can enter a hibernation-like hypometabolic state. Because hypometabolism resistance is the key function of torpor, QIH may possibly contribute to kidney protection under ischemic conditions. To validate this hypothesis, we investigated the efficiency of kidney protection during experimental circulatory arrest in normal and reduced body temperature conditions, using a transgenic mice model in which hypometabolism is induced by Q neurons activation.

## Materials and Methods

### Animals

**Figure 1:**
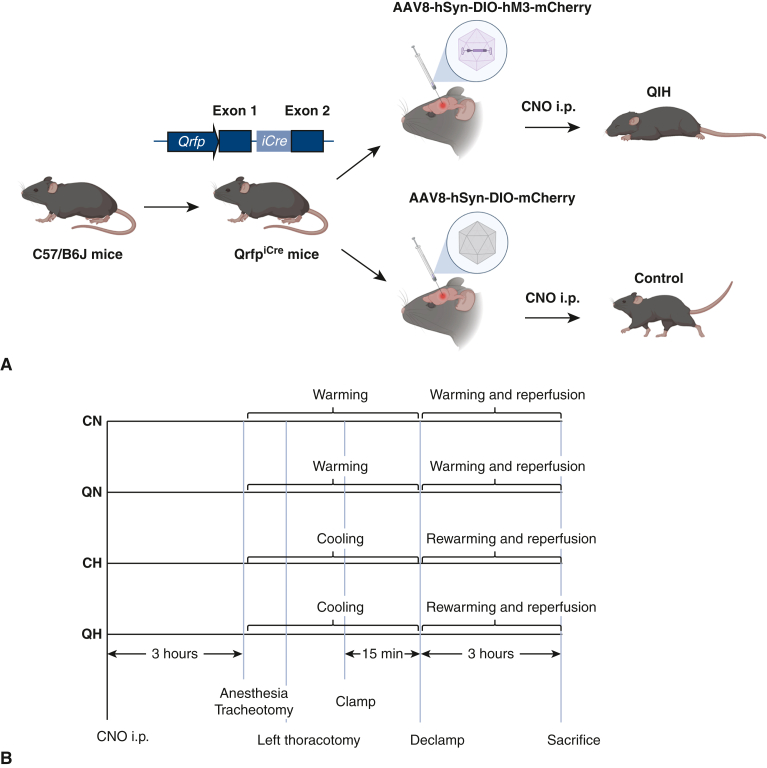

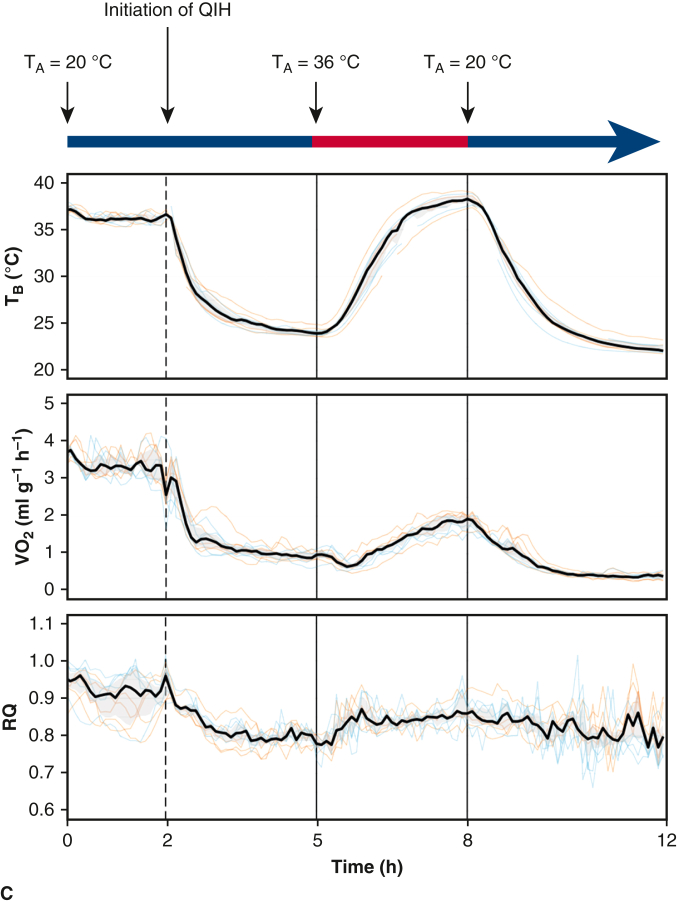
Induction of Q neurons-induced hypometabolism (*QIH*) in a mouse model. A, Schematic diagram of the preparation of QIH-ready mice and the induction of QIH. B, Surgical procedure. C, Recording of body temperature (*T*_*B*_) and rate of oxygen consumption (*V**o*_*2*_) in accordance with ambient temperature (*T*_*A*_). *Black solid lines* indicate the median and the *colored light lines* indicate raw data from each experiment. The *lower and upper edge of the shadowed area* denotes the first and third quartile. *AAV8*, Adeno-associated virus 8; *hSyn*, human synapsin; *DIO*, double-floxed inverted ORF; *hM3*, human M3 muscarinic; *Qrfp*, pyroglutamylated RFamide peptide; *iCre*, codon-improved Cre recombinase; *CNO*, clozapine-N-oxide; *i.p.*, intraperitoneal administration; *RQ*, respiratory quotient; *CN*, control normothermia; *QN*, quiescence-inducing neurons-induced hypometabolism-ready normothermia; *CH*, control hypothermia; *QH*, quiescence-inducing neurons-induced hypometabolism-ready hypothermia.

We prepared 36 male C57BL/6J background mice in which Q neurons specifically express codon-improved Cre recombinase (iCre), a Cre recombinase (Qrfp^iCre^ heterozygous mice), aged 16 to 23 weeks and of 24.4 to 36.2 g body weight as previously reported.^15^ The Qrfp^iCre^ heterozygous mice were generously provided by Dr Takeshi Sakurai, University of Tsukuba and bred at RIKEN Center for Biosystems Dynamics Research. We prepared QIH-ready mice as previously reported.^15^ In brief, the Qrfp^iCre^ heterozygous mice were anesthetized with isoflurane and positioned in a stereotaxic frame (David Kopf Instruments) to inject adeno-associated virus (AAV) at the hypothalamus to introduce designer receptors exclusively activated by designer drugs. AAV8-hSyn-DIO-hM3D (Gq)-mCherry (Addgene; 44361-AAV8, Lot #v78582, 2.1 × 10^13^ genome copies/mL) 0.3 μL was injected at the position of anterior-posterior (+0.38 mm), medial-lateral (±0.30 mm), and dorsal-ventral (–5.25 mm). Virus was injected at a controlled rate of 0.1 μL/min using a Hamilton needle syringe. When this virus infects the cells expressing iCre, the cell will start expressing hM3Dq, which can excite the neurons at the existence of clozapine-N-oxide (CNO) (designer receptors exclusively activated by designer drugs system). In this case, intraperitoneal injection of CNO will induce QIH through excitation of Q neurons.^15^^,^^16^ For the control, we injected AAV8-hSyn-DIO-mCherry (Addgene, 50459-AAV8, Lot #v48443, 2.3 × 10^13^ genome copies/mL) to express mCherry to the Q neurons. Because mCherry cannot respond to CNO, administrating CNO will not induce QIH in the control animals (Figure 1, *A*). Both QIH-ready and control mice are produced from the same strain Qrfp^iCre^ heterozygous mice, which has the C57BL/6J background. After at least 3 weeks from the virus injection, all mice were intraperitoneally injected with CNO to test the capability of QIH. Mice that showed QIH were used as QIH-ready for further ischemia induction surgery. Control mice were also intraperitoneally injected with CNO after the AAV injection. Mice were divided into 4 groups: QIH-ready normothermia (QN) (n = 8), QIH-ready hypothermia (QH) (n = 8), control normothermia (CN) (n = 10), and control hypothermia (CH) (n = 10) (Figure 1, *B*). The animals were prepared at RIKEN Center for Biosystems Dynamics Research. All animal experimental protocols were approved by the Animal Experimentation Committee, Kyoto University (#Med Kyo 20197) and RIKEN Center for Biosystems Dynamics Research (#A2022-08).

### Evaluation of the Rate of VO_2_ in Mice Under QIH

The body temperature of mice under QIH decreases because of induced hypometabolism.^15^ To evaluate whether the metabolic condition of mice under QIH is affected by external control of body temperature maintained at normothermia, the rate of VO_2_ in QIH mice was evaluated in accordance with the change of body temperature (T_B_) regulated by the ambient temperature (T_A_) (Figure 1, *C*). We prepared 5 QIH-ready mice aged from 17 to 22 weeks and housed in a temperature-controlled chamber (LP-400P-AR, Nippon Medical & Chemical Instruments). We recorded T_B_ using a telemetry temperature sensor (TA11TA-F10; DSI) and VO_2_ using a respiratory-gas analyzer (ARCO-2000 mass spectrometer; ARCO System). We kept the animals at T_A_ 20 °C (0 hours) and induced QIH by intraperitoneal injection of CNO 2 hours later (2 hours). After 3 hours from the initiation of QIH, we changed T_A_ to 36 °C for 3 hours (5-8 hours), then decreased T_A_ to 20 °C until 48 hours. We repeated the experiment in the same animals 2 times in a month interval (10 experiments in total).

### Surgical Procedure

**Figure 2:**
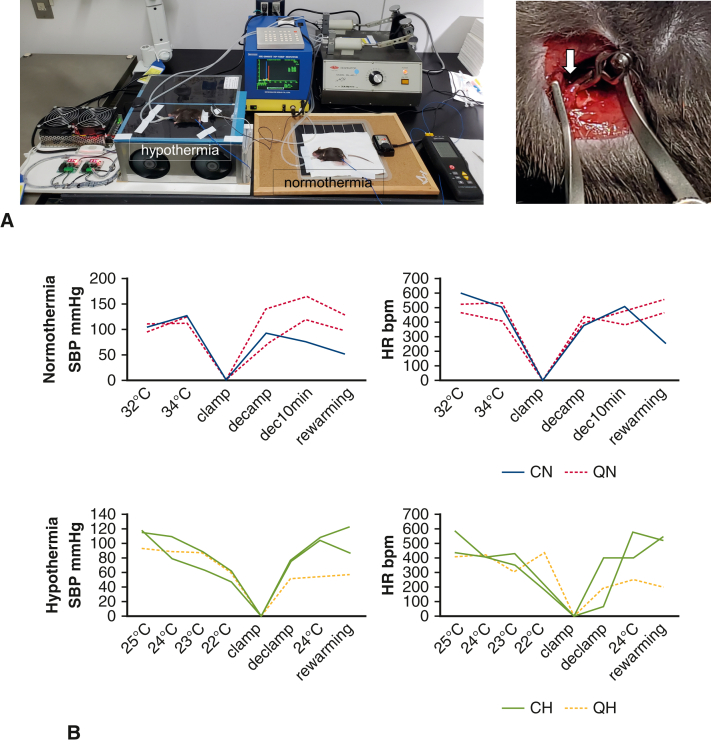

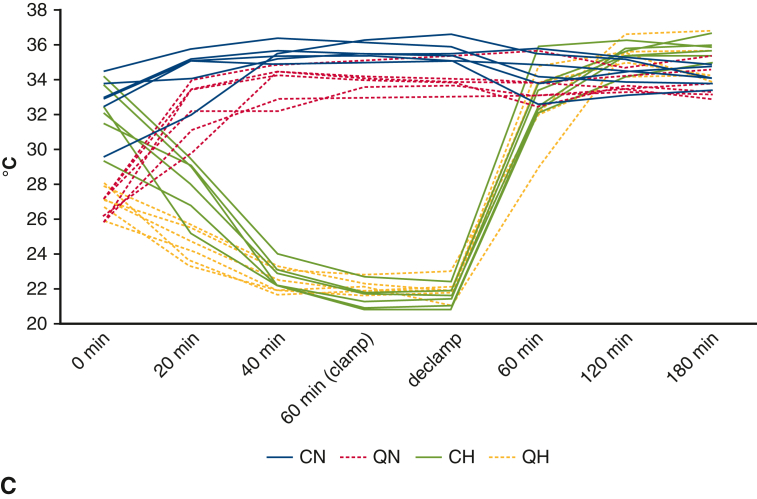
Ischemia model. A, *Left*, Setup of the surgery. Temperature control devices control the left mouse in hypothermia and the right mouse in normothermia, respectively. *Right*, Surgical view of the aortic crossclamping. The *white arrow* indicates the clamping site of the descending thoracic aorta. B, Hemodynamic parameters before, during and after aortic crossclamp. C, The change of body temperature in all mice (n = 24). *SBP*, Systolic blood pressure; *HR*, heart rate; *CN*, control normothermia; *QN*, quiescence-inducing neurons-induced hypometabolism-ready normothermia; *CH*, control hypothermia; *QH*, quiescence-inducing neurons-induced hypometabolism-ready hypothermia.

QIH-ready and control mice were transferred to Kyoto University and acclimated for 1 to 7 days with free access to food and water. The experimental protocol is indicated in Figure 1, *B*. On the day of surgery, mice were injected with CNO (1 mg/kg) intraperitoneally 3 hours before anesthesia. For tracheal intubation, mice were anesthetized with 3% to 5% isoflurane, followed by a ventral midline incision on the trachea and then tracheal intubation with a 22-gauge intravenous catheter (Terumo SR-OT2225C). Both lungs were mechanically ventilated with a tidal volume of 0.6 mL and a rate of 120 breaths per minute in room air. Vecuronium (0.1 mg/kg) and butorphanol (5 mg/kg) were intraperitoneally injected and inhalation of isoflurane ceased simultaneously. A thermometer probe was inserted into the abdominal cavity, and a custom-made temperature control device (Alice Co Ltd) controlled the body temperature modified from a previous report.^17^ In the normothermic groups, the intraperitoneal temperature was maintained at 35.0 ± 1.3 °C, whereas in the hypothermic groups, the intraperitoneal temperature was lowered until it reached 21.5 ± 1.3 °C and was maintained at a low level by the temperature control device (Figure 2, *C*). Each mouse was placed in right lateral position. An approximately 1-cm transverse thoracic incision was made and muscle on the third rib was cut, exposing the lateral pleura. A wound retractor (17003-03; F.S.T, North Vancouver, British Columbia, Canada) was used to open the incision and expose the descending thoracic aorta. After administration of heparin sodium (200 IU/kg), the descending aorta was clamped by the aortic clip (Sugita Clip No. 96, 07-940-96) for 15 minutes. Hypothermic mice were rewarmed immediately after declamping. Body temperature of each mouse was finally maintained at approximately 35 °C. After 3 hours, all survived mice were humanely put to death. Whole blood was collected from the heart and unilateral kidney was excised. The surgical procedure is shown in Video 1.

### Measurement of Blood Flow

For the measurement of blood flow before, during and after aortic clamp, we used a nonpreheating, noninvasive blood pressure, and heart rate monitor (MK-2000ST; Muromachi Kikai Co. Ltd). Using a cuff pulse sensor attached to tail arteries, we detected and measured the blood flow pattern using photoplethysmography. We measured the blood flow in 6 rats (CN:1, QN:2, CH:2, CN:1) from the induction of anesthesia to the time of death (Figure 2, *B*).

### Measurement of Serum Biochemical Parameters

Blood samples were collected and centrifuged at 10,000 rpm for 10 minutes to obtain serum. The concentration of serum neutrophil gelatinase-associated lipocalin (NGAL) and cystatin C were measured using the Mouse NGAL ELISA Kit (R&D Systems) and Mouse Cystatin C ELISA Kit (Bio Vendor R&D) according to the manufacturer's instruction.

### Histologic Examination

The excised kidneys were fixed in 10 w/v% neutral-buffered formalin, sectioned, stained with periodic acid-Schiff and photographed using a digital microscope (Biorevo BZ-9000; Keyence). Tubular injury was defined as the tubular epithelial brush border loss, lucency, flattening, loss of nuclei, and intraluminal debris/cast. Injury was scored with a semiquantitative scale; 0 = no tubular injury; 1 = 10% or less; 2 = 10% to 25%; 3 = 26% to 50%; 4 = 51% to 75%; and 5 = more than 75% of tubules injured at the cortex.^18^^,^^19^

### Statistical Analysis

The values are presented as mean ± SD. Multiple comparisons between groups were performed by 1-way analysis of variance followed by Tukey as post hoc. Statistical analyses were performed with JMP Pro 12 (SAS Institute Inc).

## Results

### Evaluation of the Rate of Vo_2_ in Mice Under QIH

As shown in Figure 1, *C*, T_B_ and Vo_2_ decreased after the initiation of QIH (5 hours). The T_B_ recovered to normal 3 hours after changing T_A_ from 20 to 36 °C (8 hours). However, the Vo_2_ at 8 hours did not recover to the level at the initiation of QIH (2 hours). These results indicate that QIH decreases the oxygen demand of organs independent of body temperature.

### Mouse Ischemia Model

In the present study, we performed ischemia induction of the lower body of mice by clamping the descending thoracic aorta, mimicking clinical aortic surgery as previously reported^20^^,^^21^ (Figure 2, *A*). As preliminary experiments, we tested several clamping duration times and found that the survival rate of the mice was extremely reduced when the clamping time was longer than 20 minutes (survival rate = 44% (4 out of 9) with a 30-minute clamp; 50% (1 out of 2) with a 20-minute clamp); therefore, we fixed our protocol to a 15-minute aortic clamping time. The survival rate was 78% (28 out of 36; 2 in CN, 3 in CH, 2 in QN, and 1 in QH died during surgery). Four animals are excluded from the experiment due to technical failure of the procedure (failure in aortic clamping) (2 in CN, 1 in CH, and 1 in QH). Finally, we evaluated 24 mice (n = 6 for each group).

We checked the blood flow of the tails of each mouse to evaluate the lower body perfusion during the procedure (Figure 2, *B*). The blood flow almost disappeared during the clamp of the aorta, which immediately recovered after de-clamping. The changes in T_B_ in all the mice are indicated in Figure 2, *C*. T_B_ in QIH mice were lower than those in non-QIH mice before aortic crossclamping, indicating successful QIH.

### Histopathologic Assessment for Renal Tubular Injury

**Figure 3:**
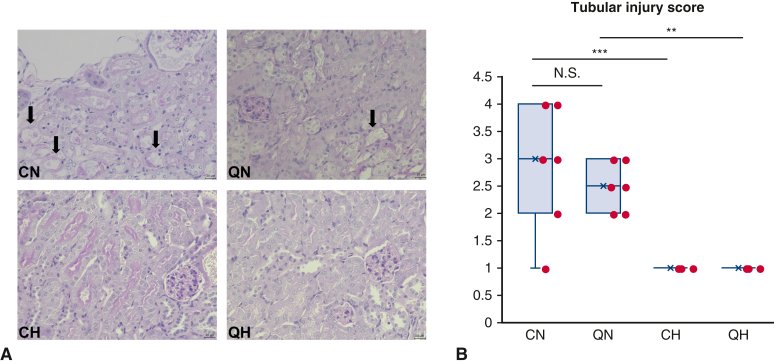
Histopathological change of the kidney. A, Representative periodic acid-Schiff staining. *Black arrows* indicate tubular injury (the tubular epithelial brush border loss, lucency, flattening, loss of nuclei, and intraluminal debris/cast). B, Tubular injury score (n = 6 per each group). All values are shown as mean ± SD. Scales bar = 20 μm in panel A. The *lower and upper borders of the box* represent the lower and upper quartiles (25th percentile and 75th percentile). The *middle horizontal line* represents the median. The *lower and upper whiskers* represent the minimum and maximum values of nonoutliers. *N.S.*, Not significant; *CN*, control normothermia; *QN*, quiescence-inducing neurons-induced hypometabolism-ready normothermia; *CH*, control hypothermia *QH*, quiescence-inducing neurons-induced hypometabolism-ready hypothermia. ∗∗*P* < .01. ∗∗∗*P* < .001.

The degree of renal tubular injury investigated by histologic analyses is shown in Figure 3. Severe tubular injury, indicated by tubular epithelial brush border loss, flattening, and loss of nuclei was observed in normothermic groups (Figure 3, *A*). Semiquantitative assessment of renal tubular injury demonstrated a significant increase in tubular injury scores in normothermic groups compared with hypothermic groups (QN vs QH: 2.5 ± 0.55 vs 1.0 ± 0; *P* = .0021, CN vs CH: 3.0 ± 1.1 vs 1.0 ± 0; *P* < .001) (Figure 3, *B*). These results indicate that hypothermia protected the kidney from sublethal renal tubular damage.

### Biomarkers for AKI

**Figure 4:**
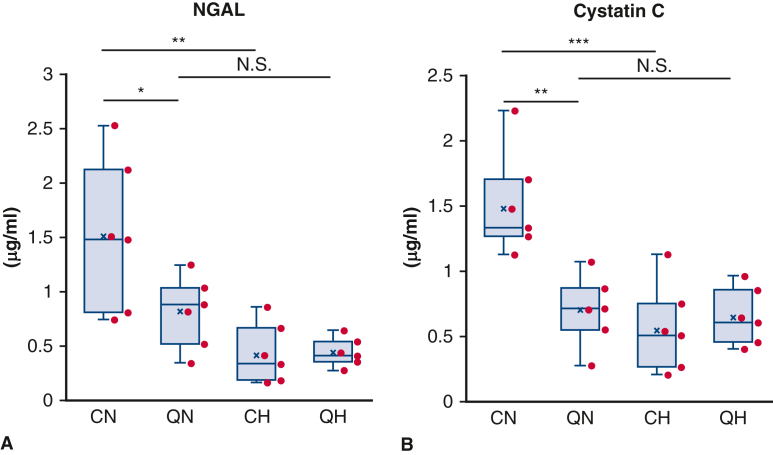
Serum biochemical markers for acute kidney injury. A, Serum neutrophil gelatinase-associated lipocalin (*NGAL*) (n = 6 per each group). B, Serum cystatin C (n = 6 per each group). All values are shown in mean ± SD. The *lower and upper borders of the box* represent the lower and upper quartiles (25th percentile and 75th percentile). The *middle horizontal line* represents the median. The *lower and upper whiskers* represent the minimum and maximum values of nonoutliers. *N.S.*, Not significant; *CN*, control normothermia; *QN*, quiescence-inducing neurons-induced hypometabolism-ready normothermia; *CH*, control hypothermia *QH*, quiescence-inducing neurons-induced hypometabolism-ready hypothermia. ∗*P* < .05. ∗∗*P* < .01. ∗∗∗*P* < .001.

Serum NGAL level and serum cystatin C level are shown in Figure 4. NGAL, measured in urine or plasma, is a highly sensitive biomarker for structural renal injury. QN exhibited lower NGAL levels than CN (CN vs QN: 1.51 ± 0.71 vs 0.82 ± 0.32; *P* = .0414, CN vs CH: 1.51 ± 0.71 vs 0.42 ± 0.27; *P* = .001). Meanwhile, there was no significant difference between QN and QH (QN vs QH: 0.82 ± 0.32 vs 0.44 ± 0.13; *P* = .411).

Serum cystatin C is a highly specific biomarker of renal function. QN exhibited lower cystatin C levels than those in CN (CN vs QN: 1.48 ± 0.39 vs 0.71 ± 0.26; *P* = .0015, CN vs CH: 1.48 ± 0.39 vs 0.55 ± 0.33; *P* = .0002). There was no significant difference between QN and QH (QN vs QH: 0.71 ± 0.26 vs 0.65 ± 0.22; *P* = .987). These results indicate that QIH, although in normothermia, protected kidneys from sublethal renal tubular damage.

## Discussion

**Figure 5:**
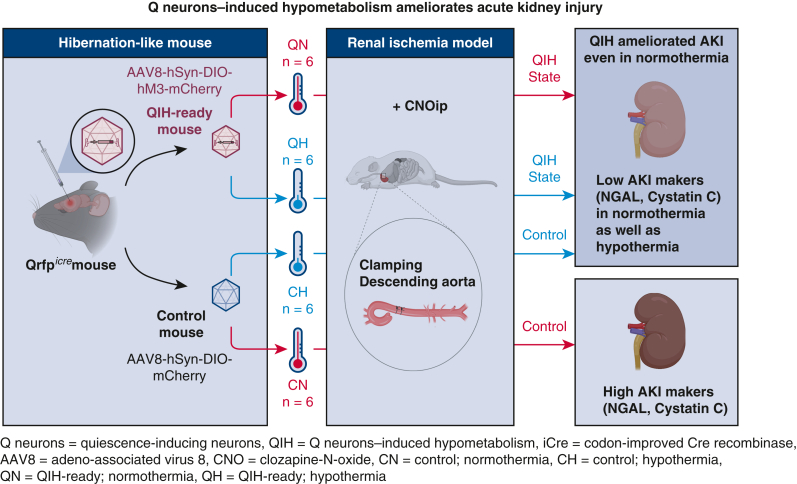
Quiescence-inducing neurons (*Q neurons*)-induced hypometabolism (*QIH*) ameliorates acute kidney injury (*AKI*). The outline of the present research is shown. *QN*, QIH-ready normothermia; *AVV8*, adeno-associated virus 8; *hSyn*, human synapsin; *DIO*, double-floxed inverted ORF; *hM3*, human M3 muscarinic; *CNO*, clozapine-N-oxide; *QH*, QIH-ready hypothermia; *NGAL*, neutrophil gelatinase-associated lipocalin; *Qrfp*, pyroglutamylated RFamide peptide; *iCre*, codon-improved Cre recombinase; *CH*, control hypothermia; *CN*, control normothermia.

In the present study, we revealed that QIH ameliorates AKI and dysfunction induced by the ischemia-reperfusion even in normothermic mice. QIH reduced kidney damage that was confirmed by histologic evaluations and the reduction of serum biomarkers levels indicating early kidney damage (Figure 5).

In the histopathology evaluations, the extent of the injury of proximal convoluted tubule was lower in hypothermic groups compared with those in normothermic groups, which may indicate the effect of kidney protection by the reduction of body temperature. On the other hand, biomarkers for early kidney damage were almost equivalent in QIH groups regardless of the body temperature, indicating that QIH, a hibernation-like hypometabolism, may confer an equivalent level of kidney protection in the situation of circulatory arrest as that mediated by hypothermia. Hibernation is a hypothermic, hypometabolic, adaptive response engaged by animals known as hibernators to reduce metabolic demand.^22^ There are several reports that have investigated the ischemia-reperfusion injury of hibernators. Lindell and colleagues^23^ evaluated liver ischemia-reperfusion injury in a liver transplantation model using squirrels as a hibernator and rats as a nonhibernator and found that the squirrel model showed significantly less elevation of serum level of lactate dehydrogenase compared with the rat model; indicating that the induction of hypometabolism in hibernators would be advantageous for organ protection. It is possible that the hibernation-like energy demand reduction of QIH realized the amelioration of ischemic kidney damage in the present study.

Passive organ protection and hypometabolism induced by hypothermia have been applied broadly and traditionally not only in the field of cardiovascular surgery, but also in various fields of medicine such as ambulatory medicine and organ transplantation, with research reports that date to the 1940s.^4^^,^^24^ QIH is a brand-new, promising approach to achieving active hypometabolism induced by the activation of Q neurons located in the hypothalamus.^15^ In a transgenic mouse model ready for QIH, the hibernation-like state can be maintained for several days. The QIH reduces body temperature by approximately 10 °C associated with massively reduced tissue oxygen consumption, heart rate, respiratory rate, and activities, resembling the passive hypometabolism induced by hypothermia. However, a hibernation-like state in the QIH is an active induction of hypometabolism, which may confer additional effects in organ protection from that in hypothermia and can be applied in not only the field of cardiovascular surgery but also in various medical fields. It would be ideal for cardiovascular surgeries requiring circulatory arrest if sufficient organ protection is achieved even with normal body temperature by the QIH, which may avoid bleeding tendency mediated by hypothermia,^8^^,^^9^ and possible kidney injury mediated by DHCA, which is reported in a rabbit cardiopulmonary bypass model.^25^ A method to induce hibernation in humans is expected for cardiovascular surgery requiring circulatory arrest, and extrapolating mouse QIH to humans is highly anticipated. Manipulation of specific neurons at the hypothalamus is required for QIH. Stimulating neurons directly in the brain may be a choice, although such invasive approaches should be avoided in patients receiving anticoagulation therapy who are undergoing cardiovascular surgery. Pharmacologic excitation of Q neurons is yet another approach to inducing human QIH. However, Q neurons-specific molecular targets, such as membrane receptors or channels, are unknown. We think that reproducing QIH is not the only answer to achieving human hibernation because brain is merely the center of torpor but not the frontline of hypometabolism. The peripheral tissues or organs during torpor should have gained resistance to hypometabolism. Therefore, among our research goals at the top of the list would be to induce torpor in humans pharmacologically by targeting the peripheral organs but not the brain.

For the evaluation of renal ischemia in mice, it is common to clamp renal arteries and veins en bloc.^26^ Although our model, in which we clamped thoracic descending aorta, would be more invasive than renal vessels clamp models, we selected this method as a model relevant to human aortic surgeries requiring the circulatory arrest of lower body.^20^^,^^21^ In the present study, we anesthetized mice with muscle relaxant and analgesic reagent under the approval of the institutional review board, which would have attenuated the effect of the reduction of body temperature and metabolic activities mediated by general anesthesia itself and enabled us to evaluate the effect of QIH on the reduction of metabolic activities more directly.

There are several limitations in the present study. First, we might have not completely eliminated the effect of anesthesia on the reduction of metabolic activities, although we had arranged the methods of anesthesia. Further, larger animals would be less susceptible to passive T_B_ change influenced by T_A_ under general anesthesia. Second, it would be preferable if we could evaluate kidney injury at a longer duration of reperfusion after ischemia considering that the majority of the research evaluated kidney injury with a longer duration of reperfusion.^27^^,^^28^ In the present study, we conducted intraoperative management using a tracheostomy that prohibited us from returning the mice after procedures for longer observation after the surgery. Third, we could not evaluate physiological parameters indicating AKI such as urine output because of relatively short observation period of renal reperfusion. Physiological end point data are anticipated in future preclinical experiments with larger animals and longer observation period, and subsequent clinical studies.

## Conclusions

In the present study, we found that QIH partly ameliorated AKI in a mouse ischemia model even in normothermia. QIH might be a promising approach to achieving sufficient kidney protection without DHCA in the future.

### Conflict of Interest Statement

The authors reported no conflicts of interest.

The *Journal* policy requires editors and reviewers to disclose conflicts of interest and to decline handling or reviewing manuscripts for which they have a conflict of interest. The editors and reviewers of this article have no conflicts of interest.

## Supplementary Data

Video 1 Surgical procedure. Video available at: https://www.jtcvs.org/article/S2666-2736(22)00373-4/fulltext.
