# Supplementary figures and images for: Quiescence-inducing neurons-induced hypometabolism ameliorates acute kidney injury in a mouse model mimicking cardiovascular surgery requiring circulatory arrest
Source: JTCVS Open. 2022 Nov 8;12:201–10. doi: 10.1016/j.xjon.2022.11.001 (PMC9801336; doi:10.1016/j.xjon.2022.11.001)

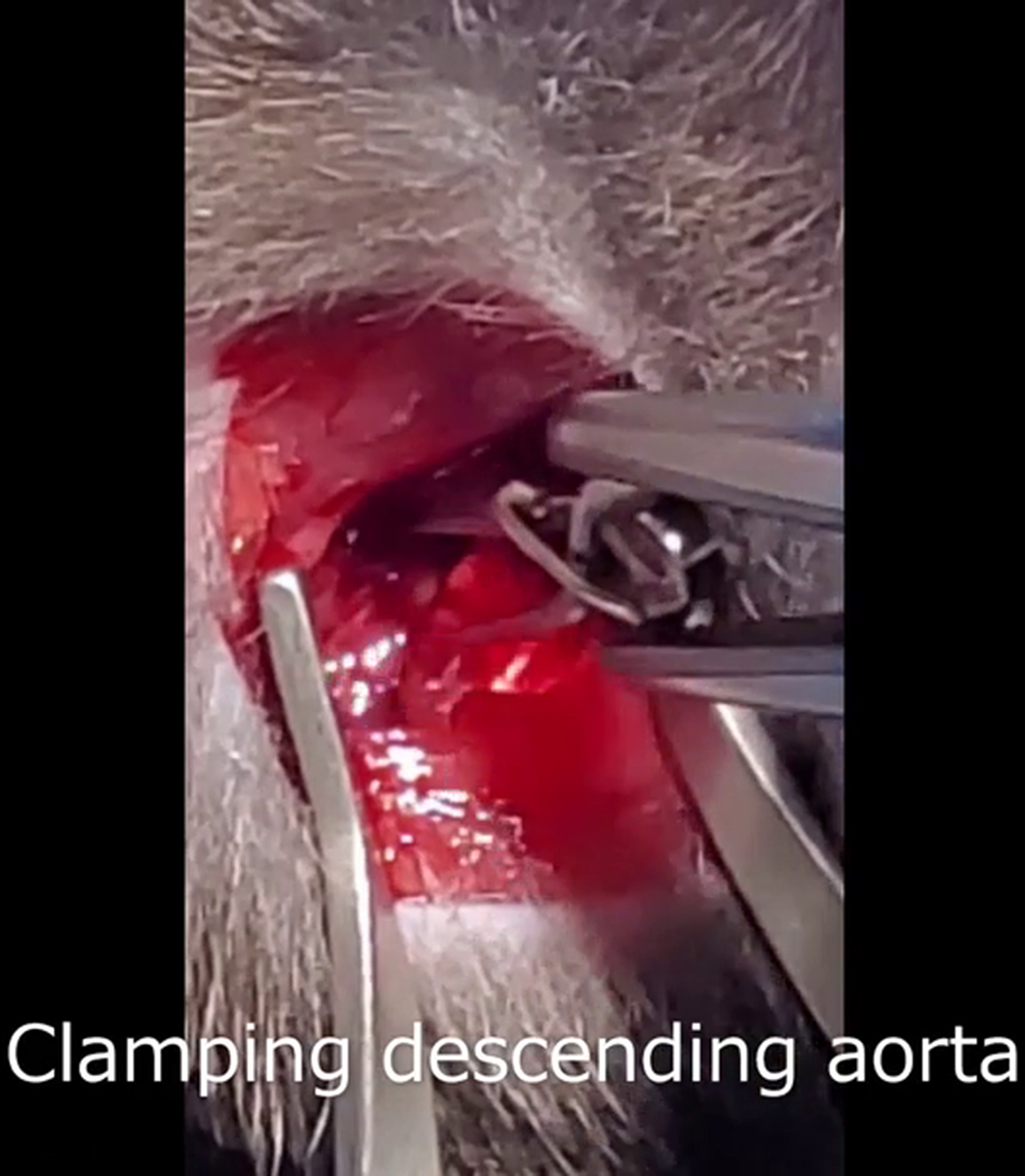

Supplement: Video 1 — Surgical procedure. Video available at: https://www.jtcvs.org/article/S2666-2736(22)00373-4/fulltext. [file fx3.jpg]
